# Mitochondrial dysfunction and oxidative stress in aging and cancer

**DOI:** 10.18632/oncotarget.9821

**Published:** 2016-06-05

**Authors:** Anna V. Kudryavtseva, George S. Krasnov, Alexey A. Dmitriev, Boris Y. Alekseev, Olga L. Kardymon, Asiya F. Sadritdinova, Maria S. Fedorova, Anatoly V. Pokrovsky, Nataliya V. Melnikova, Andrey D. Kaprin, Alexey A. Moskalev, Anastasiya V. Snezhkina

**Affiliations:** ^1^ Engelhardt Institute of Molecular Biology, Russian Academy of Sciences, Moscow, Russia; ^2^ National Medical Research Radiological Center, Ministry of Health of the Russian Federation, Moscow, Russia; ^3^ A.V. Vishnevsky Institute of Surgery, Moscow, Russia; ^4^ Moscow Institute of Physics and Technology, Dolgoprudny, Russia

**Keywords:** oxidative stress, mitochondrial dysfunction, ROS, aging, cancer, Gerotarget

## Abstract

Aging and cancer are the most important issues to research. The population in the world is growing older, and the incidence of cancer increases with age. There is no doubt about the linkage between aging and cancer. However, the molecular mechanisms underlying this association are still unknown. Several lines of evidence suggest that the oxidative stress as a cause and/or consequence of the mitochondrial dysfunction is one of the main drivers of these processes. Increasing ROS levels and products of the oxidative stress, which occur in aging and age-related disorders, were also found in cancer. This review focuses on the similarities between ageing-associated and cancer-associated oxidative stress and mitochondrial dysfunction as their common phenotype.

## INTRODUCTION

Mitochondria are important ancient organelles present in nearly all eukaryotic cells. They play an essential role in energy metabolism [1] and other cellular processes such as the β-oxidation of fatty acids [2], maintaining proper concentration of mitochondrial matrix calcium [3], amino acids metabolism [4], heme- and iron-sulfur (Fe-S) cluster biogenesis [5, 6], control of cell death including apoptosis [7–9], steroid synthesis [10], and hormonal signaling [11, 12].

Mitochondria consist of outer and inner membranes separated by an intermembrane space. The outer mitochondrial membrane (OMM) contains porins, which mediate the exchange of small molecules and information between mitochondria and the rest of the cell [13]. The inner mitochondrial membrane (IMM) encloses the matrix space and has numerous invaginations called cristae. The number of cristae per mitochondrion is related to the energy requirement for the vital functions of certain cell type as well as the number of mitochondria per cell. Cristae extend the available working space of the inner membrane surface area [14]. IMM is enriched in the proteins involved in mitochondrial fusion, transport of nuclear-encoded proteins, oxidative phosphorylation (OXPHOS), iron-sulfur cluster biogenesis, protein synthesis and transport of mtDNA-encoded proteins [15, 16].

Mitochondrial genome is a small circular DNA molecule. There are multiple copies of mitochondrial DNA (mtDNA) in the matrix of each mitochondrion. Replication of mtDNA is not related to cell cycle and may be performed many times [17]. This leads to generation of mtDNA mutations by replication errors in addition to ones due to accumulated damage [18]. The point mutations or rearrangements of mtDNA are mainly related to the OXPHOS dysfunction and cause a variety of human mitochondrial diseases as well as mutations in nuclear genes involved in the maintenance of mitochondria [19–25].

The mitochondrial dysfunction is known to be associated with aging, age-related diseases and cancer. Herein, we review current studies in the area to show that oxidative stress, as the cause or consequence of defect mitochondrial function, could be a common phenomenon in these pathologies.

## MITOCHONDRIA AND REACTIVE OXYGEN SPECIES

The first paper on free radical theory was published by Denham Harman in 1956 [26]. This theory suggests that free radicals, produced by mitochondria as by-products of their normal metabolism, later go on to attack cell constituents. A year after the publication of Harman's paper, Mills described a factor that coacted with the glutathione to protect the hemoglobin in the erythrocyte from oxidative breakdown. It was termed glutathione peroxidase (GPX) and further was described as the main enzyme involved in hydrogen peroxide detoxification [27–29]. In 1969 McCord and Fridovich discovered the anti-free radical enzyme superoxide dismutase (SOD), which was widely distributed within mammalian organisms [30]. Following this discovery, a number of studies demonstrated that mitochondria isolated from different sources (e.g., cow and pigeon heart [31–33], rat liver, heart, and brain [34–38], and yeast [39]) could generate hydrogen peroxide. Finally, Harman proposed that the mammalian lifespan depends on the genetic regulation of oxygen utilization rate, and suggested the Mitochondrial Free Radical Theory of Aging (MFRTA) [40].

**Figure 1:**
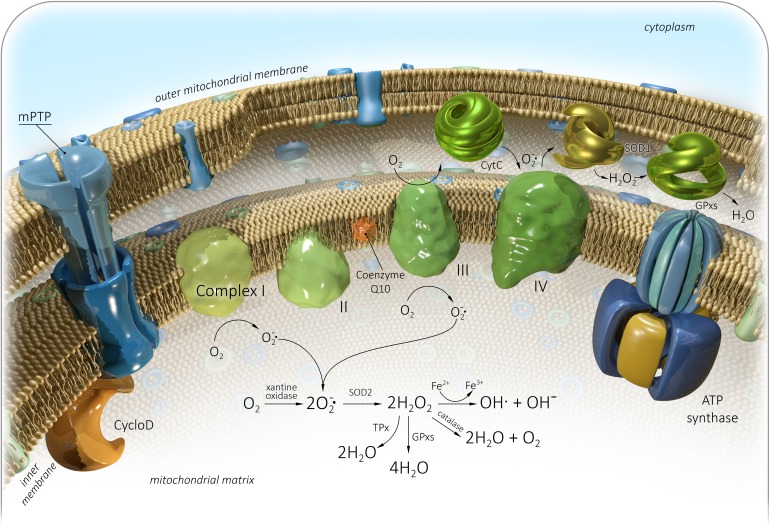
Generation of mitochondrial reactive oxygen species (mtROS) Complex I - NADH dehydrogenase, II - Succinate dehydrogenase, III - bc1 complex, IV - Cytochrome C oxidase, V - ATP synthase, Q - Ubiquinone, Cyt C - Cytochrome C, Cyclo D - Cyclophilin D, mPTP - Mitochondrial permeability transition pore, SOD - Superoxide dismutase, GPxs - Glutathione peroxidase, TPx - Thioredoxin peroxidase. See text for details.

A product of mitochondrial oxidative metabolism is highly reactive and unstable oxygen, which can oxidize many molecules and form reactive oxygen species (ROS) [41]. ROS are generated intracellular in different compartments through multiple mechanisms (Table 1). Mitochondrial-derived reactive oxygen species (mtROS) include singlet oxygen (O2), superoxide anion (O2•−), hydrogen peroxide (H2O2), nitric oxide (NO•), hydroxyl radical (OH•), and hydroxyl ion (OH-). Initially, oxygen is converted to a superoxide anion with xanthine oxidase (XO) or mitochondrial respiratory chain complexes I (NADH dehydrogenase) and III (bc1 complex) [42–45]. Complex III produces a superoxide anion in both the matrix and the intermembrane space [46]. The concentration of these complexes in IMM varies with organism, tissue, state, age or hormonal status. The superoxide anion is then converted to hydrogen peroxide by SOD. Hydrogen peroxide can be detoxified to water and oxygen with glutathione peroxidase, catalase (CAT) or thioredoxin peroxidase (TPx) [42, 47]. It can be also converted to hydroxyl radical and hydroxyl ion *via* the Fenton reaction (Figure 1) [48].

**Table 1:** Major intracellular sources of reactive oxygen species (ROS)

Reactive oxygen species	Intracellular sources	Compartment
Singlet oxygen (O2)	Fenton reaction Lipid peroxidation chain reactions Haber-Weiss reaction Superoxide Dismutase (SOD)-mediated reaction Catalase-mediated reaction Glutathione peroxidase-mediated reaction Xanthine oxidase (XO)-mediated reaction	Mitochondria Cytosol Peroxisomes Nucleus Plasma membrane Endoplasmic reticulum Lysosome All membranes
Hydroxyl radical (OH•)	Proton-catalyzed decomposition of peroxynitrite Fenton reaction Haber-Weiss reaction Decomposition of ozone (O3) Beckman-Radi-Freeman pathway	Mitochondria Cytosol Endoplasmic reticulum Lysosome
Hydrogen peroxide (H2O2)	Superoxide dismutase (SOD)-mediated reaction NADPH oxidase-mediated reaction Cytochrome P450-mediated reaction Xanthine oxidase (XO)-mediated reaction Monoamine oxidases (MAO)-mediated reaction Peroxisomal fatty acid oxidation Flavin adenine dinucleotide (FAD)-mediated reaction Antibody-catalyzed water (H2O) oxidation Electron-transfer flavoprotein pathway	Mitochondria Cytosol Peroxisomes Plasma membrane Endosomes Endoplasmic reticulum Lysosome Nucleus
Superoxide anion (O2•−)	Fenton reaction NADH/NADPH oxidase (NOX)-mediated reaction Xanthine oxidase (XO)-mediated reaction Lipoxygenase pathway Cyclooxygenase pathway Cytochrome P450 monooxygenase reaction Mitochondrial oxidative phosphorylation Electron-transfer flavoprotein reaction Hemoglobin auto-oxidation (within erytrocyte) Nitric oxide synthases (NOS)-mediated reaction	Mitochondria Cytosol Plasma membrane Peroxisomes Nucleus Endoplasmic reticulum
Hypochlorous acid (HOCL) and related species (HOBr, HOI, and HOSCN)	Eosinophil peroxidase (EPX)-mediated reaction (within eosinophil granulocytes) Myeloperoxidase (MPO)-dependent oxidation (within neutrophil granulocytes)	Cytosol Endoplasmic reticulum Lysosome Vacuole Plasma membrane Mitochondria Nucleus
Hydroxyl ion (OH-)	Fenton reaction Haber-Weiss reaction Hydroperoxide (ROOH) decomposition	Mitochondria Cytosol Endoplasmic reticulum Lysosome
Peroxide (O2•2−)	Peroxide is unstable molecule. Hydrogen peroxide is more stable one that is formed as described above.	Mitochondria Cytosol Peroxisomes Plasma membrane Endosomes Endoplasmic reticulum Lysosome Nucleus
Ozone (O3)	Ozone (O3) is unstable molecule generated during antibody catalyzed oxidation of H2O to H2O2	Cytosol
Nitric oxide radical (NO•)	Nitric oxide synthases (NOS)-mediated nitrite (NO2-) reduction Xanthine oxidase (XO) reducing nitrates and nitrites	Mitochondria Cytosol Peroxisomes Endoplasmic reticulum Plasma membrane Nucleus
Peroxynitrite (ONOO-)	Fenton reaction Rapid reaction of singlet oxygen (O2) and nitric oxide radical (NO•) The reaction of hydrogen peroxide (H2O2) with nitrite (NO2−)	Mitochondria Cytosol Lysosome Endoplasmic reticulum Nucleus Peroxisomes
Peroxyl radical (ROO•/RCOO•) (also denoted Lypid peroxyl radical (LOO•))	Lipid peroxidation chain reactions Synthesis of eicosanoids Hydroperoxide (ROOH) decomposition induced by heat or radiation ROOH reaction with transition metal ions and other oxidants capable of abstracting hydrogen	Cytosol Plasma membrane Peroxisomes Endoplasmic reticulum Mitochondria Nucleus Lysosome All membranes
Hydroperoxy radical (HOO•)	Fenton reaction	Mitochondria Cytosol Endoplasmic reticulum Lysosome
Organic hydroperoxide (ROOH/RCOOH)	Lipoxygenase-mediated reaction Oxidation of biomolecules, including lipids, proteins and DNA Cyclooxygenase reaction Cytochrome P450 monooxygenase reaction Heme-peroxidase turnover	Cytosol Plasma membrane Nucleus Endoplasmic reticulum Mitochondria Peroxisomes Lysosome
Organic radicals (R•, RO•, R-S•)	Hydroperoxide (ROOH) decomposition induced by heat or radiation ROOH reaction with transition metal ions and other oxidants capable of abstracting hydrogen Lipid peroxidation chain reactions	Cytosol Plasma membrane Mitochondria Lysosome Peroxisomes Endoplasmic reticulum Nucleus All membranes
Carbonate Radical (CO3●-)	The reaction between peroxynitrite and CO2 Superoxide Dismutase (SOD)-mediated reaction Xanthine oxidase (XO)-mediated reaction Metal-ion catalyzed decomposition of peroxymonocarbonate (HCO4-)	Mitochondria Cytosol Peroxisomes Endoplasmic reticulum Peroxisomes Lysosome Vacuole

There is a hypothesis that the nitric oxide is produced by mitochondrial NO synthase (mtNOS). This was suggested after the detection of a high rate of NO production and functionally active mitochondrial nitric oxide synthase (NOS) in rat liver mitochondria [49–51]. However, these data were not reproduced by other laboratories, implying that the NOS enzymes are not present at physiologically relevant levels in mitochondria [52, 53]. Today, NO production by mitochondria still remains an open question [54].

## LIPID PEROXIDATION

**Figure 2:**
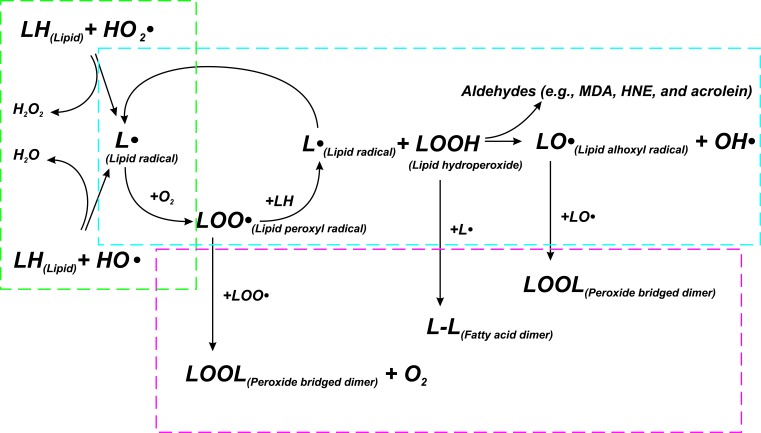
Scheme of lipid peroxidation chain reaction Lipid peroxidation chain reactions initiated by free radicals consists of three major steps (initiation, propagation, and termination), which are marked by green, blue, and red frames, respectively. LH - Lipid molecule, L• - Lipid radical, LOOH - Lipid hydroperoxide, LOO• - Lipid peroxyl radical, LO• - Lipid alhoxyl radical, LOOL - Peroxide bridged dimer, L-L - Fatty acid dimer, OH• - hydroxyl radical, HO2• - perhydroxyl radical. See text for details.

The oxidative stress leads to cell injury by three basic ways: lipid peroxidation of membranes, oxidative modification of proteins and DNA damage. Lipid peroxidation affects cell membranes and other lipid-containing structures [55]. β-oxidation of lipids is usually followed by a release of oxygen, which is reduced to water through the mitochondrial respiratory chain. At the same time, lipids can be oxidized with efficient ROS initiators, particularly hydroxyl radical and perhydroxyl radical (HO2•), forming water and a lipid radical. This initiates the reaction of lipid peroxidation, which constantly takes place in the cells. The lipid radical reacts directly with molecular oxygen and produces a lipid peroxyl radical. The lipid peroxyl radical is not a very stable molecule and can combine with another adjacent fatty acid to form a lipid hydroperoxide and different lipid radicals, or it can react with itself. Lipid hydroperoxide can be also broken down into a lipid alhoxyl radical and a hydroxyl radical. The lipid radicals formed at the previous stage can react with oxygen to produce another lipid peroxyl radical, and so on. Thus, this process is called “chain reaction of lipid peroxidation” (Figure 2). The main intermediate products of the reaction are lipid hydroperoxides (LOOHs). They can disturb membrane structure, an being dangerous for cells [56].

## PODUCTS OF LIPID PEROXIDATION AS COMMON MARKERS OF OXIDATIVE STRESS IN AGING AND CANCER

The major secondary products of lipid peroxidation are toxic and mutagenic aldehydes, malondialdehyde (MDA) and 4-hydroxynonenal/4-hydroxy-2-nonenal (HNE). They are considered markers of the oxidative stress [57–61]. These products have unique properties compared with ROS because the non-charged structure of aldehydes allows them to easily migrate through membranes and cytosol and, consequently, to cause far-reaching damaging effects inside or outside the cells [62, 63]. There is objective evidence that HNE and MDA can modify the amino acid residues and form stable adducts leading to protein damage [85, 86]. They can also form covalent adducts with nucleic acids, and membrane lipids. The MDA and HNE have been shown to be implicated in normal aging, age-related neurodegenerative diseases, and cancer [64–69]. Recent study showed that HNE-modified proteins (HNE-MP) were accumulated during aging *in vitro* and could be supposed to measure aging parameters. The middle-aged human fibroblasts were cultured and maintained by serial passaging throughout their proliferative lifespan. Four age points of the cells were analyzed. Aging cells showed a considerable increase in HNE-MP levels compared with young and middle-aged ones [70].

The HNE-production in the brain is induced by the amyloid-β peptide (Aβ), which plays a primary role in Alzheimer's disease (AD) pathogenesis [63]. Conversely, the preincubation of cells with HNE increased the uptake of Aβ and its intracellular accumulation. This indicates that HNE and Aβ may interact to provide potentiation of Aβ's cytotoxicity effects on neuron-like cells *in vitro* [71, 72]. HNE-crosslinking modifications accumulating in the lysosomal/proteasomal pathway and leading to protein inactivation and insolubility were detected in patients with Alzheimer's disease [73]. Immunocytochemical studies have demonstrated that pyrrole adducts formed by reacting HNE with lysine amino groups were present in neurons of patients with AD cases [74]. An increase in MDA immunoreactivity was detected in the cytoplasm of neurons and astrocytes in both normal aged and AD brains, but not in brains of young subjects [75]. Moreover, increased plasmatic levels of MDA and its correlation with age were also observed in AD patients [76–80].

Parkinson's disease (PD) is pathologically characterized by progressive destruction and death of neurons, that produce dopamine. HNE may alter dopamine uptake in rat striatal synaptosomes through binding to SH groups of the dopamine transporter and to Na+/K+ ATPase [81]. In rat striatal membranes, HNE have been registered as an effector of signaling pathway mediated by D1/D5 dopamine receptors [82]. In addition, it has been shown that HNE could modulate the activity of regulator G-protein signaling 4 (RGS4) involved in PD [83]. The concentration of HNE was increased in the cerebrospinal fluid and plasma of Parkinson's patients [84]. Furthermore, HNE-modified proteins were positively stained in more than half the nigral neurons of PD patients, and the levels of MDA were also increased. The data indicate that, in Parkinson disease, oxidative stress can contribute to nigral cell death [85, 86].

**Figure 3:**
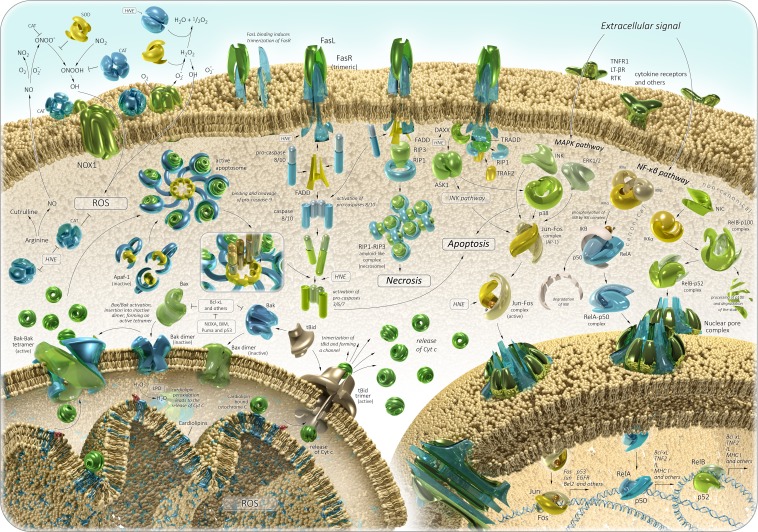
Oxidative stress in aging and cancer: signaling pathways See text for details.

Recently, it was found that the by-products of lipid peroxidation can induce carcinogenesis. Cell membranes contain a high concentration of polyunsaturated fatty acids, which are frequently subjected to peroxidation. This leads to an inhibition of growth and death of cells. The oxidation of phospholipids in the IMM can trigger the mitochondria-mediated pathway of apoptosis (Figure 3). ROS or lipid peroxidation by-products primarily react to cardiolipin molecules, the IMM phospholipids, which are bound to cytochrome c [87–89]. This induces disturbances of cytochrome c-cardiolipin interaction and dissociation of cytochrome c from the IMM [90–92]. The release of cytochrome c into the cytoplasm induces a series of biochemical reactions, resulting in caspase activation and subsequent cell death [9]. At this point, a major regulator of mitochondrion-dependent apoptosis is Bcl-2 family of proteins, which show both pro- and anti-apoptotic activities. The proteins belonging to the Bcl-2 family are bound to the OMM and can modulate its permeabilization [93]. Bax and Bak are anti-apoptotic proteins of the Bcl-2 family, which can be activated in two ways: through disturbance of their bond with anti-apoptotic proteins (e.g., Bcl-2, Bcl-xL and Mcl-1) [94], or interaction with activator proteins (e.g., BH3/tBid, Puma, BIM, NOXA, and p53), which induce their conformational changes [95–98]. Inactivated Bax proteins can be localized as monomers in the cytosol or closely associated with the OMM. Then, during the process of its activation, Bax forms homo-oligomers and inserts itself into the OMM as well as into Bak. This leads to membrane pore formation and permeabilization, which promotes the release of cytochrome c in cytosol [99]. Anti-apoptotic proteins prevent mitochondria-mediated apoptosis through their interaction with pro-apoptotic ones. The studies show that overexpression of Bcl-2 inhibits the release of cytochrome c from mitochondria and the subsequent apoptotic response is blocked. For example, HNE-induced caspase activation is suppressed in Bcl-2 transfected colorectal carcinoma cells [100]. The cytosol cytochrome c binds to the adapter protein apoptotic protease activating factor 1 (Apaf-1), and induces an apoptosome assembly in the presence of ATP/dATP. This activates pro-caspase-9 directly within the apoptosome complex [101]. Then the pro-caspase-9 is cleaved to the active caspase-9, which, in turn, activates the caspases-3, -6 and -7, leading to DNA fragmentation and cell death [101, 102]. If the cellular ATP/dATP level is depleted, the caspase activation is blocked and the cell death is re-directed from apoptosis to necrosis. The release of cytochrome *c* and apoptosome formation can be also triggered though the extrinsic pathway of apoptosis [103, 104]. Additionally, the accumulation of damage directly in mitochondria may also cause enhanced oxidant production and a cascade of degenerative events. It should be noted that HNE could be generated directly through the oxidation of mitochondrial phospholipid cardiolipin as well as other oxidation products. In this case, HNE reacts with surrounding molecules near the site of its formation, thereby promoting chain-reactions of the mitochondria-derived apoptosis again [105]. This process appears to be involved in atherosclerosis and cancer [106, 107]. Thus, it has been shown that HNE could induce mitochondria-mediated apoptosis in the pheochromocytoma (PC12) cell line and colorectal carcinoma cells [100, 108]. A statistically significant increase in MDA and HNE levels was detected in primary colorectal cancer, implying the association of colorectal carcinogenesis with serious oxidative stress [109]. Immunohistochemical staining of HNE adducts was demonstrated in animal models of liver cancer [110]. HNE treatment of MG63 human osteosarcoma cells could activate caspase-3 and altered the Bax/Bcl-2 ratio, thereby inducing cell death [111]. A recent research showed that HNE increased the growth of breast cancer cells and promoted their angiogenesis and invasion [112]. Elevated levels of MDA were observed in plasma and blood serum of patients with breast, lung, ovarian, thyroid, and oral cancer, and precancer states [113–122]. The MDA levels in patients with lung cancer correlated with the cancer stage [123]. In addition, significantly higher levels of salivary MDA were determined in squamous cell carcinoma and pre-cancer patients [124].

## LIPID PEROXIDATION PRODUCTS AND SIGNALING PATHWAYS

Several studies suggest an interesting mechanism protecting tumor cells against superoxide anion-mediated apoptosis by the expression of membrane-associated catalase. Tumor cells, as known, generate extracellular superoxide anions with the participation of NADPH Oxidase 1 (NOX1). NOX1 are multi-subunit enzyme complexes localized in cell membrane and required for the reduction of molecular oxygen. On the one hand, extracellular superoxide anions are needed for the regulation of cancer cell proliferation and for the maintenance of their transformed state. On the other hand, extracellular superoxide anions can induce intercellular apoptosis in transformed cells [125, 126]. As a result, they are selectively eliminated. The ROS signaling by the HOCl and NO/peroxynitrite pathways are involved directly in this apoptosis-derived cell death [127–129]. In the transformed cell, extracellular superoxide anions spontaneously react with hydroxyl ions (H-). They could be also driven by SOD forming an unstable hydrogen peroxide. It is used as a substrate to generate exogenous HOCl by the dual oxidase (DUOX)-coded peroxidase (POD) domain. Then HOCl can interact with superoxide anions, generating a hydroxyl radical, which penetrates intracellular space, induces lipid peroxidation, and consequently promotes the mitochondrial-derived apoptosis. However, tumor cells express the membrane-associated catalase, which can efficiently decompose H2O2 directly after its generation. Moreover, SOD can play a co-modulatory protective role through partial inhibition of HOCl/superoxide anion interactions. SOD-derived generation of H2O2 could be also compensated by interrelation of SOD with a catalase-mediated protective effect. Additionally, in the presence of high concentrations of H2O2, compared with POD, HOCl can react with H2O2 to block the HOCl signaling [130].

NO/peroxynitrite signaling pathway modulating tumor cell death consists of the following: (1) within cells, NO synthase promotes arginine-derived NO synthesis, (2) NO passes through the cell membrane, (3) the formation of peroxynitrite extracellular interaction of NO with superoxide anion and the subsequent reactions lead to the generation of nitrogen dioxide (NO2) and hydroxyl radical, (4) these reactions initiate the lipid peroxidation and apoptosis [131]. Nevertheless, the membrane-associated catalase can protect the tumor cell by preventing peroxynitrite generation and NO oxidation [126]. Moreover, the inhibition of NO/superoxide anion interactions by SOD, as in the previous case, modulates the negative effect on apoptosis pathway. Interestingly, it has been shown that high concentrations of HNE may inactivate membrane-associated catalase, leading to tumor cell death through mitochondria-derived apoptosis and consequently bestowing anti-cancer effects (Figure 3) [126, 132].

In a multitude of studies, it has been shown that HNE and its protein adducts can regulate Nuclear factor-kB (NF-κB) and activator protien 1 transcription factor (AP-1) transcription factors, thereby being responsible for the expression of many genes (Figure 3). It is well known that NF-kB signaling pathway is involved in normal aging, age-related diseases, and cancer [133–138]. NF-κB is a family of proteins, which play a key role in regulating the expression of genes which are responsible for immunity, apoptosis, and cell cycle. NF-κB family consists of five proteins: p65 (RelA), RelB, c-Rel, p105/p50 (NF-κB1), and p100/52 (NF-κB2), which associate with each other to form distinct active NF-κB dimers. In the cytosol, NF-κB dimers in inactive form anchored by IκB are found. There are two ways to activate the NF-kB-induced gene transcription. These are triggered by cytokines TNFα and IL-1 (canonical signaling) or antigen receptors CD40 and BAFF (non-canonical/alternative signaling). There are the activation of IKK complex (IKKα, IKKβ, and IKKγ) and the phosphorylation of IκB proteins, which are in interaction with p50-RelA subunit, with cytokines in the course of canonical signaling pathway. This reaction leads to degradation of IkB proteins, and a release of the active p50-RelA NF-κB dimer. NF-kB non-canonical signaling consists of the following: NIK (NF-κB-inducing kinase) induces the activation of IKKα, which phosphorylates p100 NF-κB subunit of p100-RelB NF-κB dimer directly in cytosol. This leads to the generation of an active p52-RelB NF-κB dimer. In both cases, the active NF-κB dimer can translocate to the nucleus and induce the transcription of target genes. NF-κB target genes include regulators of apoptosis (Bcl-XL and IAPs), cytokines (TNFa, IL-1, IL-6, and IL-12), cyclins and growth factors (G-CSF and M-CSF), immunregulatory proteins (VCAM, ICAM, and MHC I), and others [139, 140]. Many different kinases can phosphorylate and activate the IKKα and IKKβ subunits of the IKK complex: glycogen synthase kinase 3β (GSK3β), protein kinases B, R and C (PKB, PKR, and PKC), mitogen-activated type 3-protein kinase 7 (MAP3K7), p38 MAP kinases or c-Jun N-terminal kinases (JNKs) [141]. Thus, it has been shown that HNE could differently regulate PKC isozymes, MAPK (mitogen-activated protein kinase), and JNK in a dose-dependent manner and be involved in NF-kB activation (Figure 3) [142–145].

AP-1 is a heterodimeric/homodimeric complex composed of members of the JUN, FOS, ATF, and MAF families. The combination of protein in the AP-1 complex determines its target genes that play a major role in differentiation, proliferation, and apoptosis. Important examples are Bcl2 family of proteins, EGFR, p53, CD44, and proliferin. AP-1 complex has been shown to be involved in tumorigenesis. Two components of AP-1, c-JUN, and c-FOS, are well known as oncoproteins, but in some cases they can suppress tumor formation [146]. The regulation of AP-1 activity occurs by many ways: differential expression of single AP-1 components, interactions with ancillary proteins, and transcriptional and post-translational regulation. The last way consists in MAPK pathways activation (ERK1/ERK2, JNK/SAPK, and p38) by several external stimuli. This leads to induction of FOS and JUN gene transcription and the formation of further AP-1 complexes. It has been shown that HNE can regulate AP-1 complex generation through induction of FOS and JUN gene expression or activation/inhibition of MAPK pathways [108, 147–150].

Moreover, HNE has been shown to regulate the Fas/Fadd-dependent pathway of apoptosis (Figure 3). Fas is a protein belonging to the TNF-receptor superfamily. The Fas receptor (FasR) contains a death domain, which is a protein interaction module. FADD is an adaptor molecule, which contains a death domain and a death effector domain. FADD can interact with members of the TNF-receptor superfamily and mediates cell apoptotic signals. Binding of FasR with Fas ligand (FasL) induces its trimerization. This leads to interaction with adaptor molecule FADD, which has already been recruited by the receptor-associated death proteases (pro-caspases-8 and -10) through the death effector domain. Thereby, the death-inducing signaling complex I (DISC complex I) is formed. Activated caspase-8 can stimulate the caspase-3 signaling in two ways: (1) caspase-8 cleaves the Bcl-2 interacting protein (Bid), which alters the mitochondrial membrane permeabilization or induces activation of Bak. It triggers cytochrome c release, thereby activating the mitochondrion-dependent pathway of apoptosis; (2) caspase-8 directly activates caspase-3, -7 or -6, leading to the apoptotic DNA fragmentation and cell death [151]. In another case, FasR can recruit death-associated protein (DAXX), which further binds with apoptosis signal-regulating kinase 1 (ASK1) and activates it [152, 153]. Then ASK1 induces JNK/SAPK and p38 MAPK pathways [154–156]. The interaction of FasR with receptor-interacting protein kinase 1 (RIP1) was supposed to be a component of DISC complex II [157]. DISC complex II, depending on the combination of signaling proteins, can promote apoptosis, necrosis, NF-kB, JNK/SAPK and p38 MAPK pathways. A study by Chaudhary and colleagues has demonstrated that HNE could induce a Fas-mediated apoptosis in HepG2 cells. They demonstrate that an exposure of HepG2 cells to sublethal concentrations of HNE promoted the export of DAXX from the nucleus to cytoplasm and facilitated Fas-DAXX binding. In its turn, it activated ASK1, JNK and caspase-3, leading to cell death [158]. The same data were obtained in Jurkat and HLE B-3 cells [159, 160]. Generally, it has been shown that the expression and functions of Fas can be modulated by HNE in a time- and concentration-dependent manner and binding of HNE with Fas was essential for the execution of apoptosis. The involvement of HNE in p53 apoptosis pathway in HepG2 cells was also determined. Treatment of HepG2 cells with HNE resulted in the induction of p53 expression, its phosphorylation, and activation of downstream targets Bax and p21 [158]. Actually, it was shown that HNE treatment increased the expression of p53 family proteins and their targets in the SK-N-BE neuroblastoma cell line [161]. A recent study demonstrated that HNE treatment of SH-SY5Y cell culture induced the abnormal expression of apoptotic markers (p53, Bax and caspase-3) and led to neuronal cell death [162].

As previously mentioned, AD pathogenesis is triggered by the progressive accumulation of the amyloid-β peptide in the form of extracellular amyloid plaques in human brain. Aβ results from a cell surface receptor and transmembrane precursor amyloid protein (APP). Within lipid rafts, APP cleaved by β-secretase leads to a generation of a membrane bound carboxyl (C)-terminal fragment (CTF-β). CTF-β needs to be cleaved by γ-secretase to form Aβ and the amyloid precursor protein intracellular domain (AICD). Then Aβ molecules self-aggregate into soluble oligomers. It was discovered that the levels of this soluble fibrillar oligomers were increased in the brains of AD patients and correlated with the disease [163]. Moreover, Aβ oligomers were found in both intracellular and extracellular species [164–166]. Thus, Aβ oligomers can contribute to AD pathology *via* different mechanisms, including the induction of neurotoxicity, the formation of insoluble fibrillar amyloid-β aggregates, and the facilitation of tau pathology [167, 168]. Tau is the microtubule-associated protein (MAP), which stabilizes neuronal microtubules and is located, mainly, in the axons of neurons in central nervous system. An abnormal hyperphosphorylation of tau protein in brain leads to the generation of neurofibrillary tangles (NFTs) known as a hallmark of Alzheimer's disease [169, 170]. It has been shown that HNE bound directly to normal tau and induced the tau Alz50 epitope involved in hyperphosphorylation of tau protein and neurofibrillary tangle formation in Alzheimer's disease [171, 172]. A study by Liu and co-authors confirmed these findings to show that an antibody against neurofibrillary tangles recognized tau in brains of AD patients more effectively after HNE-treatment, but only when tau was in the phosphorylated state [173]. The immunocytochemical study previously showed that HNE treatment of cultured rat hippocampal neurons caused a moderate increase in the basal levels of tau phosphorylation, and prevented tau dephosphorylation [174].

## OXIDATION PROTEIN-ADDUCTS

Many studies have reported that HNE- and MDA-protein adducts were associated with Alzheimer's disease progression. They show that Aβ oligomers can insert into the cell membranes and promote lipid peroxidation. This led to generation of by-products of lipid peroxidation such as MDA and HNE. Moreover, in brain tissue, lipid peroxidation-derived aldehydes can be also formed in many ways mentioned above. Then MDA and HNE can react and covalently modify many critical proteins such as amyloid-β peptide, collapsing response mediator protein 2 (CRMP2), neuronal glucose transporter 3 (GLUT3), neuropolypeptide h3, carbonyl reductase (NADPH), lactate dehydrogenase B (LDHB), heat shock protein 70 (HSP70), elongation factor Tu (EF-Tu), elongation factor 1 alpha (eIF-α), and manganese superoxide dismutase (MnSOD). It promotes neuronal cell impairment and Alzheimer's disease pathogenesis [175–178]. For example, the immediate reaction of HNE with the Aβ peptide leads to the formation of more toxic diffusible Aβ-oligomers and insoluble aggregates [179]. The HNE-amyloid-β peptide adducts have an increased affinity for lipid membranes and tendency to form amyloid fibrils. Thus, the stimulation of lipid peroxidation by Aβ results in its own modification and accelerates amyloidogenesis [180, 181].

Elevated levels of several HNE-modified proteins of energy metabolism, including alpha-enolase (ENO1), phosphoglycerate kinase 1 (PGK1), pyruvate kinase (PK), mitochondrial ATP synthase α chain (ATP5A), malate dehydrogenase (MDH) and triosephosphate isomerase (TPI), were detected in mild cognitive impairment (MCI) patients associated with a risk for Alzheimer's disease. Some of these oxidatively modified proteins are enzymes involved in glycolysis. These are ENO1, PGK1, TPI, and PK.

It is well known that brain is one of the greatest consumers of glucose, and glycolysis is required for normal functions of one. Alpha-enolase is a housekeeping enzyme, which catalyzes the hydrolysis dehydration of 2-phospho-D-glycerate (2-PGA) to phosphoenolpyruvate (PEP) in the penultimate step of glycolysis in cytoplasm. Many studies demonstrate that ENO1 is subjected to oxidative modification, which can be accompanied by decreasing its activity in different pathological conditions such as aging [182], Alzheimer's and Parkinson's diseases [183–186], Huntington's disease [187], and cancer [188, 189]. However, ENO1 does not directly affect ATP production in spite of its glycolytic function. Actually, recent studies reported that ENO1 has also a lot of non-glycolytic functions [190–194] and might be involved in more than just metabolic processing of glucose [195].

Phosphoglycerate kinase 1 is an enzyme of the glycolytic pathway regulated by hypoxia-inducible factor-1α (HIF-1α). It catalyzes the conversion of 1,3-biphosphoglycerate (1,3-BPG) to 3-phosphoglycerate (3PG) in glycolysis. HNE can both react directly with PGK1 to form HNE-PGK1-adducts and promote a decrease in PGK1 expression [196]. The altered expression and conformation of PGK1 is correlated with cellular senescence and cancer [197–200]. Furthermore, a recent study has revealed that oxidative damage of PGK1 was markedly increased in aged human frontal cortex in progressive supranuclear palsy [201]. Decreased levels of PGK1 were detected even in the hippocampus of aged rats under beneficial effects of caloric restriction [202].

Pyruvate kinase catalyzes the last step of glycolysis leading to the generation of ATP and pyruvate. PK has a key role for energy homeostasis in brain tissues [203]. It has been revealed that the increasing activity of pyruvate kinase was followed by enhancement of glucose-dependence of brains in aged rats [204]. At the same time, reduction of pyruvate kinase activity, mediated by free radicals, was found in rat cerebrum [205]. Pyruvate kinase could also submit Aβ-induced oxidative modifications in the process of AD pathology [206]. In tumor cells, PK is converted to a less active dimer form PKM2, which is a major regulator of cancer metabolism. Moreover, PKM2 is associated with caspase-independent cell death [207]. This is confirmed by the fact that, in cancer cells, caspases can downregulate primary regulators of pyruvate kinase activity, particularly, phosphoserine [208].

Triosephosphate isomerase is an essential enzyme for glycolysis and gluconeogenesis, which catalyzes the interconvertible isomerization of glyceraldehyde 3-phosphate (GAP) and dihydroxyacetone phosphate (DHAP) [209]. TPI deficiency is a severe glycolytic defect that contributes to progressive neurological dysfunction [210]. Actually, it was demonstrated that the inhibition of TPI might lead to neurodegeneration [211]. Lower TPI activity is detected in aged senescence-accelerated mice brain models, which show early cognitive impairment [212]. Also, modified TPI interacts with tau protein, inducing an intraneuronal aggregation, thereby contributing to the acceleration of AD progression [213]. Conversely, tau can have an effect on TPI, triggering its functional loss and subsequently facilitating neurodegenerative disease development [214]. Many studies elucidated that TPI was also involved in tumorigenesis and anti-drug resistance of cancer cells [215–217].

There are two isoenzymes of malate dehydrogenase: mitochondrial and cytoplasmic ones. Mitochondrial MDH catalyzes the reaction of reversible malate oxidation to oxaloacetate using the reduction of NAD+ to NADH. Conversely, cytoplasmic MDA reduces oxaloacetate to malate, oxidizing NADH to NAD+. Then malate enters into mitochondria from cytoplasm where it can be utilized by mitochondrial MDH. These reactions are components of the TCA cycle and gluconeogenesis from pyruvate. It is known that a large part of glucose molecules utilized by the brain is produced *via* gluconeogenesis. The altered activity of MDA is associated with Parkinson's disease [218], Alzheimer's disease [219], schizophrenia [220], and cancer [221, 222].

Mitochondrial ATP synthase α chain plays a crucial role in the activity of the entire electron transport chain and subsequently in ATP production. Thus, a failure of ATP5A leads to the loss of the whole ATP synthase (complex V) activity. This event, coupled with the changes in complex I, III, and IV, may result in impairment of mitochondrial ATP production, the leakage of electrons from their carrier molecules, and further ROS generation. Schägger and Ohm previously reported ATP-synthase deficiencies in Alzheimer's disease [223]. In addition, decreased activity of ATP-synthase was detected in brains of patients with late-stage AD [224].

These results confirm the contribution of mitochondrial dysfunction to AD progression [176]. Moreover, the altered activity of energy metabolism enzymes on the whole is one of general stages in the progression of age-related diseases and cancer.

## OXIDATIVE DNA DAMAGE

Oxidative DNA damage appears to be critical for aging, age-related diseases and cancer [225]. ROS and products of lipid peroxidation can have an effect on both genomic and mitochondrial DNA, leading to various types of DNA damage: double- and single-strand breaks, intra- and interstrand DNA crosslinks, DNA-adduct formation, DNA base and deoxyribose modifications. Subsequently, replication of damaged DNA before repairing results in DNA mutations and genomic instability [226]. The DNA double-strand breaks (DSBs) are the most dangerous impairment. They cause severe genetic mutations leading to various disorders and tumor progression [227–229]. The single-strand breaks (SSBs) are less harmful for cells if they are repaired in time. If they are not repaired rapidly, chromosomal SSBs also result in serious lesions and may contribute to many human diseases [230]. Moreover, DNA replication of SSBs can potentially lead to DSBs formation. It has been shown that a transient increase in DSBs may induce Aβ-derived DNA damage caused by a synaptic dysfunction and being involved in the pathogenesis of Alzheimer's disease [231–233]. In addition, larger numbers of SSBs and DSBs were observed in the brains of PD patients [234].

8-oxoGuanine (8-OHG) is one from the multiple oxidation products generated in DNA through dG oxidation. 8-OHG can join erroneously to adenine to make G-T and C-A replacements in genome. The nucleoside form of 8-OHG is 8-oxo-2′-deoxyguanosine (8-OHdG), which has been proposed as an indicator of oxidative DNA damage *in vivo* and *in vitro* [235, 236]. 8-OHdG further can be subjected to keto-enol tautomerism to favor the oxidized product 8-oxo-7,8-dihydro-2-deoxyguanosine (8-oxodG), which is also commonly used as a marker of oxidative DNA damage [237, 238]. Many studies reported a direct correlation between 8-OHG formation and carcinogenesis [239]. Altered levels of 8-OHG/8-OHdG demonstrated an association with pathogenesis of Alzheimer's disease, amyotrophic lateral sclerosis, Down's syndrome, Parkinson's disease, normal aging, and cancer [237, 240–248]. For example, Ames and colleagues have shown the age-dependent accumulation of 8-OHdG in DNA from various rat organs [249]. Increased levels of 8-OHdG and OH8Gua, one more marker of oxidative DNA damage, in senescent human diploid fibroblast were shown [250]. DNA adducts derived from dC oxidation, 2′-deoxycytidine (dC)-5-hydroxy-2′-deoxycytidine (OH5dC), 5-hydroxy-2′-deoxyuridine (OH5dU), and 5,6-dihydroxy-5,6-dihydro-2′-deoxyuridine (dUg), were also detected in organs of different aged rats at levels similar to those of 8-OHdG [251]. It may contribute to spontaneous mutagenesis, leading to cancer and aging. A study of 5,6-hydroxy-5,6-dihydrothymine/dihydrothymidine (dTg), a product of dT oxidation, in mouse and monkey urine revealed a correlation between the specific metabolic rate of a species and the urinary outputs of dTg. The urinary dTg levels from mice were higher than those from monkey [252]. These data demonstrated that increased metabolic rate and oxidative DNA damage were associated with the shorter life span typical for smaller mammals. Thus, the presence of DNA oxidation products may be related to a higher oxidative stress and lower level of some antioxidants. In this case, there is a higher level of DNA lesions, which are not completely repaired.

Mitochondria generate large amounts of ROS directly exposing mtDNA to oxidative stress. The levels of oxidative mtDNA damage are more than a half higher and more extensive compared to nuclear DNA [235]. mtDNA lacks “protective” histones and has a limited repertoire of available DNA repair pathways, therefore it is very sensitive to oxidative damage. There is a “vicious cycle theory of mitochondrial ROS production”. It consists in the following: mtDNA damage results in mitochondrial dysfunction leading to an increase in ROS production, which subsequently elevates the accumulation rate of mtDNA mutations, which will further impair respiratory chain function [253]. However, this theory is still being discussed in the field of research on aging, cancer, and oxidative stress [254–259].

There is an evidence that the accumulation of oxidative mitochondrial DNA damage during normal aging is a risk factor for the development of age-associated neurodegenerative disorders [260]. It has been shown that the frequency of point mtDNA mutations increased approximately 5-fold during an 80-year lifespan [261]. The accumulation of somatic mtDNA mutations was demonstrated to be a feature of accelerated aging in knock-in mice, expressing a proofreading-deficient version of the mitochondrial DNA polymerase G (POLG) and to promote apoptosis [262]. Another similar study confirmed these data and showed a causative link between mtDNA mutations and aging phenotypes [263]. The mtDNA damage, correlated with elevated mitochondrial ROS formation, was demonstrated to significantly contribute to age-dependent endothelial dysfunction in vessels [264]. Aliev and co-authors showed that mtDNA deletions were accompanied by increased levels of APP, 8-OHG and cytochrome c oxidase (COX), and correlated with endothelial lesions in vessels [265]. These findings explain the fact that aging-related impairment of cerebral perfusion results in brain hypoperfusion, which contributes to the development of AD and consequently to neurodegeneration [266]. Increased of mtDNA damage and 8-OHG levels, associated with reduced mtDNA content, were observed in AD brains [267–270]. The age-related increase in mtDNA damage was shown in patients with sporadic Parkinson's disease [271]. Greater accumulation of mtDNA deletions was detected in the dopaminergic neurons of substantia nigra in old rats compared to young ones [272].

In recemt years, several mutations and depletions of mtDNA were identified in different types of cancers. Mutations associated with the development of tumors were found to be present in both the non-coding and coding regions of mtDNA in patients with leukemia and various types of carcinoma [273, 274]. However, it is unclear whether the mitochondrial dysfunction is a cause or a consequence of cancer. A recent study of a cohort including 311 individuals with mitochondrial dysfunction (90% maternally inherited mtDNA mutation) has not shown an increased risk of cancer compared with the general population. However, these results do not contradict the hypothesis that secondary mtDNA alterations are formed during tumorigenesis, which can play an essential role in the further malignant transformation [275–277].

## MITOCHONDRIAL HORMESIS

The MFRTA suggests that oxidative damage is accumulated with age and drives the aging process. There is a linear dose-response relationship between the increasing amounts of ROS and the oxidative stress. However, several lines of research demonstrated the potential beneficial role of ROS as redox signaling molecules. Mitochondria are fully integrated into the cell, and any significant deficiency in mitochondrial function may trigger an adaptive nuclear response, thereby altering nuclear gene expression [278]. Thus, it has been reported that low doses of ROS exposure decreased mortality and increased stress resistance, while higher doses exerted opposite effects [279]. Moreover, the effect of antioxidants on the mitochondrial ROS signal impairs the general health and prevents the extension of lifespan [280, 281]. ROS is termed mitochondrial hormesis or mitohormesis [282]. It has been detected that mitohormesis extended the lifespan in many model organisms, including *Saccharomyces cerevisiae*, *Drosophila melanogaster*, *Caenorhabditis elegans* and mice [278, 280]. The concept of mitohormesis bases on the physiological effects of calorie and glucose restriction, reduction of specific macronutrients, and physical exercises, which are required to promote health and longevity, and the role of ROS as essential signaling molecules in these processes.

It was found that ROS influenced stress resistance and lifespan through several transcription factors, such as FOXO/DAF-16, NRF2/SKN1, and HSF-1 [279]. For instance, NF-E2-Related Factor 2 (NRF2), which is activated by ROS, can bind with the antioxidants responsive elements (AREs) and mediate mitohormesis [283, 284]. Other transcription factors, such as Forkhead transcription factors (FOXOs) and heat shock factor 1 (HSF-1), activate many genes involved in cellular stress response. HSF-1 is a major repressor of heat shock genes, which encode proteins rapidly induced after temperature stress. FOXOs regulate the transcription of superoxide dismutase and catalase genes, which encode the enzymes involved in detoxification of ROS [285, 286]. Several studies show that the mechanisms underlying extended lifespan are dependent on the AMP-activated protein kinase (AMPK) [281, 287–290]. AMPK is a cellular energy sensor, which is activated by metabolic stress. AMPK upregulates the activities of many key metabolic enzymes, thereby, compensates for the energy deficit and increases the oxidative stress resistance and survival rates [287]. It is suggested that the extension of lifespan requires activation of p38 MAP kinase, which can induce ROS formation [291, 292]. The impairment of the mTOR pathway is shown to extend the lifespan in various organisms [293–295]. The impairment of insulin/IGF-1 signaling prolongs the lifespan of mice [296]. HIF-1 responds to hypoxia by activating the transcription of many genes. It is shown that HIF-1 can down-regulate the mitochondrial activity and be responsible for lifespan extending through RNAi-mediated knockdown of several mitochondrial proteins [279, 297, 298].

It should be noted that the longevity is closely related to an increased risk of cancer and neurodegenerative disorder incidences. Thus, it is not surprising that the molecular mechanism underlying mitohormesis can be associated with age-related diseases and cancer. In support of this, many studies showed the critical role of Nrf2, HIF-1, and p38 MAP kinase in progression of neurodegenerative disease and cancer, in which oxidative stress is closely implicated [299–312].

## CELLULAR SENESCENCE AND TUMOR SUPPRESSION

The cellular senescence has been first described by Hayflick and colleagues more than 40 years ago [313, 314]. They demonstrated that normal diploid cells had a limited replicative potential. In contrast to cancer cells, normal cells at the end of their replicative life span are in a process known as cellular senescence which is characterized by irreversible cell cycle arrest, morphological changes, epigenetic modifications, lack of response to growth factors, telomere shortening and dysfunction, sustained metabolic activity and elevated DNA damage [315]. Further, cellular senescence has been supposed to be a tumor-suppressor mechanism [316]. Many studies showed the activation of the negative growth regulatory genes and proteins, which inhibit the initiation of DNA synthesis, during senescence. For example, p53 and Rb, the tumor suppressors, were shown to play a role in the regulation of cellular senescence [317]. Inactivation of p53 and Rb genes contributed to extend the proliferative lifespan of normal fibroblasts [318–320]. However, it is not sufficient for malignant transformation. Studies by Cairns revealed that at least four or five mutations were required for the transformation from normal to cancer cells [321, 322]. Using a genetic model of colorectal tumorigenesis, Vogelstein and collaborators also proposed at least four genetic alterations that could underlie tumor development [323]. The other growth inhibitory genes such as p21, p16, p33, p19, and p27 were also regarded as key effectors of cellular senescence [317, 324].

Ras and Myc oncogenes have been shown to be involved in the regulation of senescence-inducing pathways. Serrano and colleagues in 1997 demonstrated that oncogenic transformation of human diploid and mouse embryo fibroblasts by RAS resulted in a permanent cell-cycle arrest, simultaneous induction of the p53 and p16 tumor suppressor proteins, and cellular senescence. They found that oncogenic transformation of the cells by RAS required either a cooperating oncogene or the inactivation of tumor suppressors. However, escape from RAS-induced arrest by disruption of p53 or p16/Rb pathways may lead to cell transformation [325]. In contrast to RAS, Myc overexpression resulted in apoptosis in primary cells. This effect could be mediated by activation of p19^ARF^/p53 and p14^ARF^/E2F-1 pathways [326, 327]. The expression of other oncoproteins, such as Raf and MEK, also induces cell cycle arrest and activates mediators of senescence (p53, p21^Cip1^, and p16^Ink4a^) in human fibroblasts [328–330]. Jacobs and co-authors showed that overexpression of Bmi-1 oncogene induced primary mouse fibroblast immortalization and downregulated expression of the tumor suppressors p16 and p19^Arf^. Bmi-1-deficient mouse fibroblasts, in turn, showed an increased expression of p16 and p19^Arf^ and premature senescence phenotype [331].

Telomere shortening is one of the major mechanisms inducing cellular senescence and inhibiting tumorigenesis [332, 333]. However, it can trigger not only senescence response but telomere crisis as well. Progressive telomere shortening and a loss of tumor suppressor function result in a massive chromosomal instability, secondary genetic alterations and facilitate carcinogenesis [334, 335]. For example, a study of a telomerase-knockout mouse, heterozygous for mutant p53, revealed that a loss of telomere function and the consequent genomic instability could cooperate with p53 deficiency and promote tumorigenic initiation [336]. In addition, a series of studies revealed the relative roles of senescence and apoptosis induced by telomere dysfunction and p53 activation in tumor suppression [337].

mTOR pathway is involved in both senescent phenotype and cancer, which have been extensively studied by Blagosklonny and colleagues. mTOR pathway drives the process of conversion from proliferative arrest to irreversible senescence and is involved in longevity [338, 339]. On the other hand, it is activated by mutations in oncogenes such as Raf, Ras, and PI3K and inhibition of many tumor suppressor genes, including p53. However, cells with TOR-activating oncogenes are required in deactivation of cell cycle checkpoints to proliferate [340].

Senescing cells are characterized by persistent DNA-damage response (DDR) signaling, which could be induced by mitochondrial dysfunction and oxidative stress. It detects DNA lesions, signals the presence of genomic DNA damage, and promotes their repair [341]. Indeed, DDR was recognized as an anticancer mechanism leading to cell cycle arrest followed by cellular senescence or apoptosis [342, 343]. Markers of DDR, such as overexpression of p21, the activation of checkpoint kinases ATM and Chk2, the phosphorylation of histone H2AX, p53 accumulation or phosphorylation, commonly occur at the early stages of human tumors and precursor lesions [344–346]. These data indicate that, at an early stage of tumorigenesis, cells activate DDR to delay or prevent cancer. Thus, DDR can be considered yet another mechanism of tumor suppression that could be triggered by oxidative stress and in which senescence is involved.

Data from several sources show that the progression of cancer is also slower in aged individuals, and metastases are often less frequent [347, 348]. The loss in genome “plasticity” during the development and aging is a possible explanation for this decline [349]. The deregulation of chromosome recombination potential predisposes to malignant transformation, but there is a decline in it with aging. Thus, during senescence, the loss in “plasticity” protects from deviations in cell proliferation, slowing down tumor growth and metastasis in the old. Moreover, cellular senescence is also mentioned as a mechanism of reversion of tumor cells to normality [350]. In tumor cell population, there are nondividing cells with growth arrest also termed senescence [351].

Normal cells have a genetically programmed time limit for cell replication and aim to avoid excessive proliferation. There is no doubt that senescence is one of the major defense mechanisms against malignant transformation. However, it was shown that under some conditions, normal cells can “escape senescence”. The treatment of primary cells with viral oncogenes results in either death of the cells or apoptosis. At a certain frequency, cells can become immortal [352]. *In vivo*, during senescence, a lot of alterations, including oncogenic ones, can be accumulated. Moreover, senescent cells secrete factors that can promote other cell growth and tumorigenesis [353]. Thus, despite senescence is recognized as a potent tumor-suppressor mechanism it remains a risk factor for cancer.

## CONCLUSIONS

**Figure 4:**
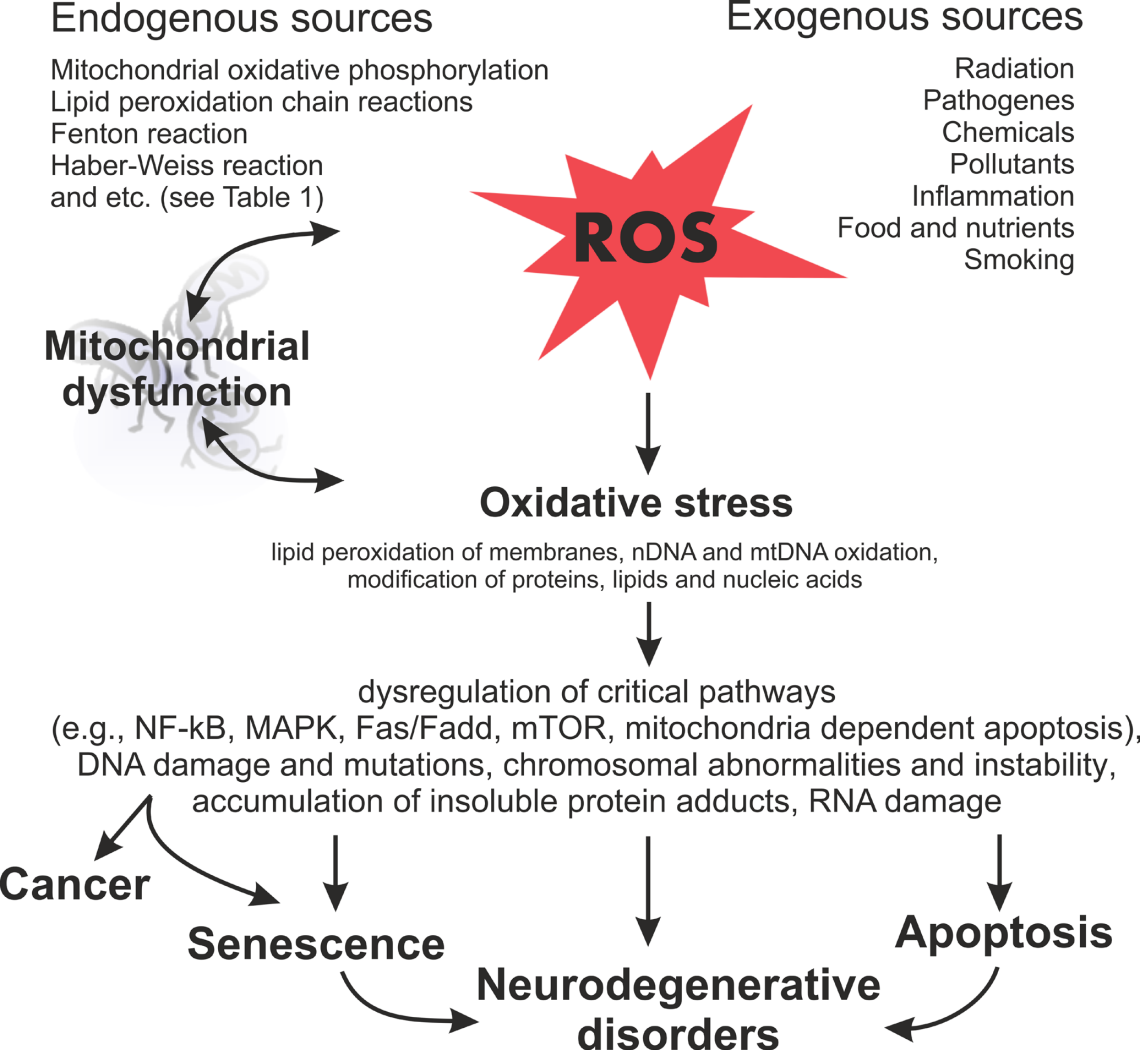
Schematic diagram illustrating the harmful effects of ROS on the cellular processes and subsequent outcomes

There is much evidence that the normal aging and carcinogenesis are multistep processes, which can be induced by ROS (Figure 4). Mitochondria generate ROS during normal metabolism. ROS, in turn, may have an effect on many important intracellular components. For example, they attack the mitochondrial membranes and mtDNA directly near the place of their formation. This leads to mitochondrial dysfunctions and more ROS production. Furthermore, ROS interacts extensively with nuclear DNA and proteins, leading to DNA damage and protein-adduct formation. ROS also interacts with lipids of cell membranes to disturb their functions. Actually, large amounts of DNA mutations or arrangements, genomic instability, impairment of protein functions and altered metabolic and signal pathways, induced by oxidative stress, have been found in cells subjected to pathological conditions, such as aging or malignant transformation. It has been demonstrated that age-related neurodegenerative disorders, such as Alzheimer's and Parkinson's diseases and different types of cancers, have similar disturbances. The energy metabolism is altered in both neurodegenerative disorders and tumors. However, in the former case, this leads to nerve cell death. In the latter case, such alteration results in tumorigenesis.

Thus, we suggest that mitochondrial dysfunction and oxidative damage inevitably occur in normal aging and can lead to age-related neurodegenerative disorders. Under various conditions, these processes may be a risk factor for cancer.
